# Three-dimensional morphology of first molars in relation to ethnicity and the occurrence of cleft lip and palate

**DOI:** 10.1371/journal.pone.0185472

**Published:** 2017-10-09

**Authors:** Sandra Echtermeyer, Philine H. Metelmann, Alexander Hemprich, Karl-Heinz Dannhauer, Karl-Friedrich Krey

**Affiliations:** 1 Echtermeyer Dental Practice, Leipzig, Germany; 2 Department of Orthodontics, Center for Dental, Oral and Craniomandibular Sciences, University Medicine Greifswald, Greifswald, Germany; 3 Department of Maxillofacial Surgery and Plastic Surgery, University Medicine Leipzig, Leipzig, Germany; 4 Department of Orthodontics, Center for Dental, Oral and Craniomandibular Sciences, University Medicine Greifswald, Greifswald, Germany; Medical University of South Carolina, UNITED STATES

## Abstract

**Objectives:**

This study aims to describe morphological peculiarities of maxillary and mandibular first molars in Europeans, Asians and Europeans with cleft lip and palate.

**Material and methods:**

Reflex microscopy was used to obtain three-dimensional morphometric landmarks from 40 models (11 Europeans and 13 Asians without cleft lip and palate, 16 Europeans with unilateral cleft lip and palate). The cases were examined using traditional morphometry and geometric morphometry, and visualized using thin-plate splines.

**Results:**

Classic morphometry showed no right/left differences in the study groups and no significant differences with regard to the cleft side in patients with cleft lip and palate. In Asians, a significantly greater mesiodistal width was found. Geometric morphometry showed an enlarged centroid size in Asians (maxilla and mandible). In cleft patients, the cleft site did not appear to impact the morphology of first molars.

**Conclusion:**

Unilateral clefting did not affect the size and shape of molars; however, characteristic ethnicity-based differences were in fact identified. The results are relevant for orthodontic treatment with preadjusted appliances, and prosthetic CAD/CAM restorations.

## Introduction

The development of molar crowns is a complex process that begins in the 28th week of gestation. The result are teeth shaped in a universal pattern with highly personalized details. In the case of a eugnathic dentition, this morphology enables a finely balanced function of static and dynamic occlusion. In a dysgnathic dentition, however, this functional balance can only be achieved through orthodontic treatment or by restoring lost tooth structure. It is therefore essential to have knowledge of the individual’s tooth shape and the average morphology of the individual’s ethnic group. The first molar (M1) plays a key role in this context [1]: Discrepancies in the mesiodistal diameter between permanent first molars on the left and right sides were described in connection with the development of asymmetric malocclusions [2].

To a large extent, tooth morphology is determined genetically [3] but can be modulated by external factors. High conformity in the tooth morphology of monozygotic and dizygotic twins [4] supports this hypothesis.

This study focuses on two hypothetical aspects that influence molar morphology:

1. Ethnicity: The influence of ethnicity on tooth form has long been described [5] but is rarely factored into current orthodontic concepts. Forensic dentistry, however, frequently uses tooth morphology to assign ethnicity [6,7].
2. Cleft lip and palate: Akcam et al. found evidence that there is a correlation between cleft lip and palate and smaller premolars [8]. However, this statement was based only on linear measurements on plaster models.

If these influences significantly change the morphology of teeth, the automatic CAD/CAM generation of restorations and orthodontic attachments should not be based on generalized algorithms.

Although the morphology of molars has long been an issue in anthropology [9], few systematic investigations have been conducted in the field of orthodontics.

### Questions

1. Are there characteristic differences in the size and shape of first molars in the maxilla (upper jaw) and mandible (lower jaw) between Europeans, Asians and Europeans with unilateral cleft lip and palate?
2. Are there differences in first molar morphology between the right and left side of the dental arch?
3. Does unilateral clefting influence the morphology of first molars?

## Materials and methods

For this retrospective study, 1000 plaster models were examined from the archives of a Department of Orthodontics.

After consulting the ethics committee of the institution, an ethics approval for this type of study was deemed unnecessary since no information was used that would allow connections to be made to private patient information. All models were analyzed anonymously and no patients were directly involved in the study. All plaster models were taken from an archive that was set up for research purposes and no plaster models were made for the sole purpose of this study.

All models were manufactured in the same dental laboratory (Alginate impressions: Tetra chromium, Kanidenta GmbH & Co. KG, Herford, Germany; Siladent Ortho Plaster, Siladent Dr. Böhme & Schöps GmbH, Goslar, Germany). Models were interrelated in the maximum intercuspal position.

The plaster models used in the investigation were obtained from patients showing mixed dentitions, representing the main orthodontic treatment age. Since abrasion of tooth structure can be considered a regular process that strongly influences morphology [10], only teeth of young people without signs of malfunction (grinding, pressing or abrasive habits) were used in the investigation.

The inclusion criteria were defined as follows:

- For the group of Europeans and the group of Asians: no patients with syndromes or other craniofacial or dental malformations or systemic diseases
- For the group of European cleft patients: unilateral complete cleft lip and palate
- Normal development of dentition and vertical adjustment of the teeth
- No hypoplastic teeth, no anomalies in tooth shape, no anomalies in number of teeth, no prominent Cusp of Carabelli; four-cusp molars in the maxilla and five-cusp molars in the mandible
- Identifiability of all measurement points
- No abrasions
- No fillings, fissure sealings or prosthetic restoration
- No previous orthodontic treatment with multibracket appliances

The use of such strict inclusion criteria meant that most plaster models were ill-suited for the study.

Based on these criteria, 40 models—11 European, 13 Asian, 16 European with unilateral cleft lip and palate, as described in Table 1 - were evaluated. The study has an 80% statistical power of detecting a difference of 0.512 (effect size) at alpha = 0.05. In general, conclusions based on the results of this study should be made with caution.

**Table 1:** Examined models.

Group	n	Male	Female	Age [year]	
				Mean	SD
European	11	7 [63%]	4 [37%]	10.7	2.41
Asian	13	11 [84%]	2 [16%]	10.5	2.21
Cleft	16	13 [81%]	3 [19%]	10.8	2.46

Owing to the small number of models that met the inclusion criteria, the unequal gender distribution was deemed acceptable in order to avoid reducing the sample size any further.

The overjet, overbite and dental arch width (Mühlberg analysis [11]) were measured to further characterize the groups (Table 2).

**Table 2:** Characterization of the groups based on orthodontic metrics.

	European		Asian		Cleft	
	Mean [mm]	SD [mm]	Mean [mm]	SD [mm]	Mean [mm]	SD [mm]
Overjet	4.2	3.41	3.1	2.27	0.8	3.34
Overbite	4.7	1.95	2.9	1.40	2.3	2.83
Anterior arch width maxilla	35.8	2.57	37.1	2.50	34.5	5.65
Anterior arch width mandible	34.7	2.11	37.1	2.16	35.2	2.92
Posterior arch width maxilla	46.1	3.01	48.2	3.39	46.9	4.19
Posterior arch width mandible	47.8	2.44	50.0	2.66	47.1	3.64
Arch length maxilla	17.9	2.69	17.8	2.75	16.3	4.60
Arch length mandible	15.6	2.11	16.4	2.20	14.6	2.72

The three-dimensional measurement of the models was performed using a reflex microscope (Reflex Measurement Ltd, London, UK) with a 15x magnification, as introduced by Scott [12].

**Fig 1:**
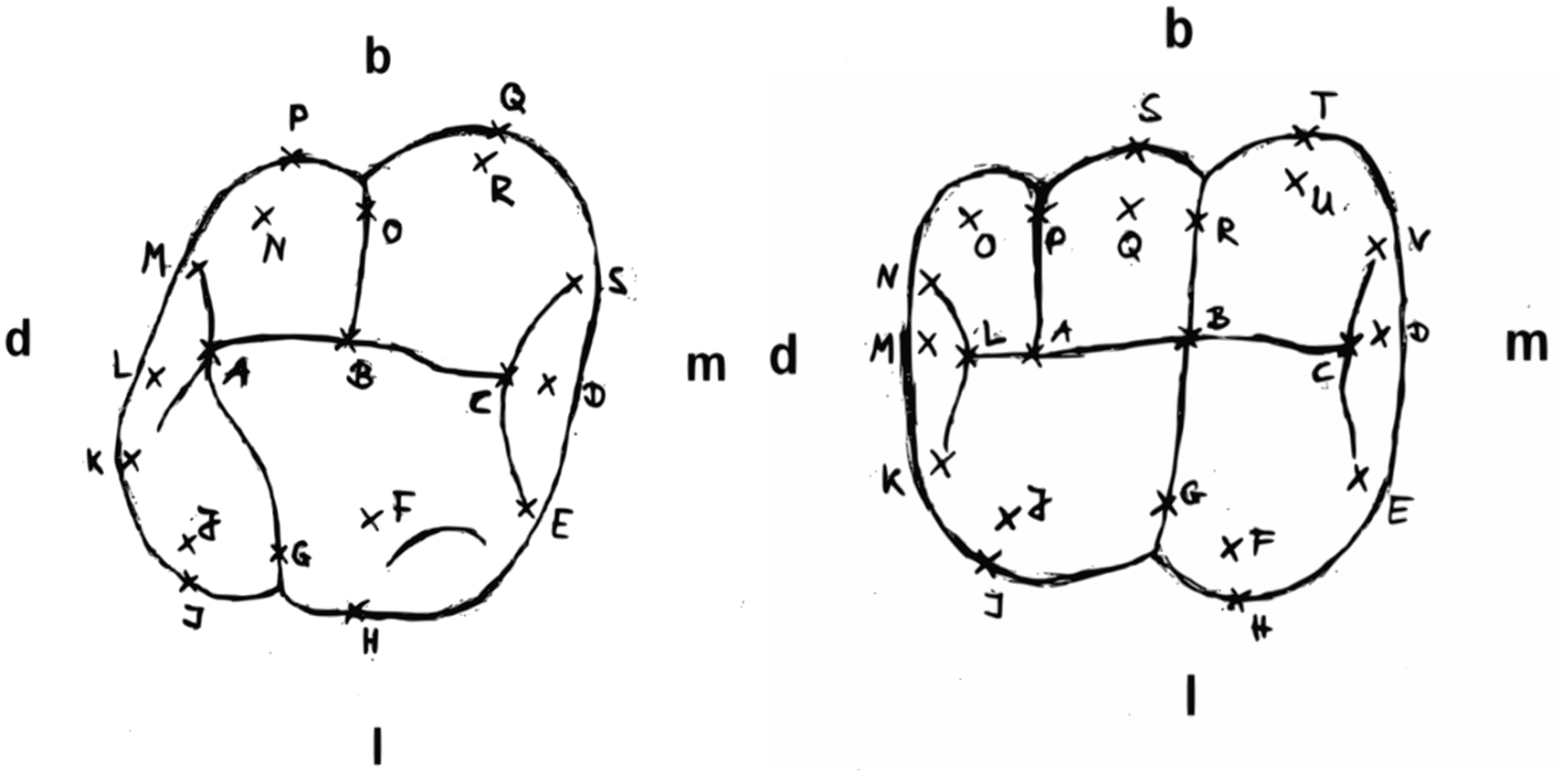
Measuring points in the maxilla (“OK”) and mandible (“UK”). Orientation: buccal (b), lingual (l), distal (d), mesial (m).

The measurement points (Fig 1, Table 3) in this investigation were selected based on earlier studies by Gómez-Robles et al. [13] and Hartmann [14].

**Table 3:** Measuring points on the molars.

Maxilla		Mandible	
Point	Description	Point	Description
A	distal fissure	A	contact point of central fissure and disto-buccal fissure
B	center of the central fossa → deepest point	B	center of the central fossa → deepest point
C	mesial fissure	C	mesial fissure
D	mesial marginal ridge (most mesial point, opposite side of mesial fissure)	D	mesial marginal ridge (most mesial point, opposite side of mesial fissure)
E	contact point of mesio-lingual cuspal slope with mesial marginal ridge	E	contact point of mesio-lingual cuspal slope with mesial marginal ridge
F	tip of mesio-lingual cusp (Paracon)	F	tip of mesio-lingual cusp (Paraconid)
G	contact point of mesio-lingual cuspal slope with disto-lingual cuspal slope	G	contact point of mesio-lingual cuspal slope with disto-lingual cuspal slope
H	maximum contour opposite to mesio-lingual cusp tip	H	maximum contour opposite to mesio-lingual cusp tip
I	maximum contour opposite to disto-lingual cusp tip	I	maximum contour opposite to disto-lingual cusp tip
J	tip of disto-lingual cusp (Metacon)	J	tip of disto-lingual cusp (Metaconid)
K	contact point of disto-lingual cuspal slope with distal marginal ridge	K	contact point of disto-lingual cuspal slope with distal marginal ridge
L	distal marginal ridge (most distal point, opposite to distal fissure)	L	distal fissure
M	contact point of distal tilt of distal-buccal cuspal slope with distal marginal ridge	M	distal marginal ridge (most distal point, opposite to distal fissure)
N	tip of disto-buccal cusp (Hypocon)	N	contact point of distal tilt of distal-buccal cuspal slope with distal marginal ridge
O	contact point of mesio-buccal cuspal slope with disto-buccal cuspal slope	O	tip of distal cusp
P	maximum contour opposite to disto-buccal cuspal tip	P	contact point of disto-buccal cuspal slope (distal) with distal cuspal slope (mesial)
Q	maximum contour opposite to mesio-buccal cuspal tip	Q	tip of disto-buccal cusp (Hypoconid)
R	tip of mesio-buccal cusp (Protocon)	R	contact point of mesio-buccal cuspal slope with disto-buccal cuspal slope
S	contact point of mesio-buccal cuspal slope with mesial marginal ridge	S	maximum contour opposite of disto-buccal cuspal tip
		T	maximum contour opposite of mesio-buccal cuspal tip
		U	tip of mesio-buccal cusp (Protoconid)
		V	contact point of mesio-buccal cuspal slope with mesial marginal ridge

The software C3D (Reflex Measurement Ltd, London, UK) was used to trace the xyz-coordinates for all measurements and to calculate the distances and angles.

Statistical analysis was performed using SPSS Statistics 19 (IBM—Armonk, NY, USA) and R 2.12.2 (http://cran.r-project.org). Palaeontological Statistics (PAST) [15,16] were employed to conduct analysis using geometric morphometry.

For the test results, p ≤ 0.05 was determined to be statistically significant (*).

### Traditional morphometry

**Fig 2:**
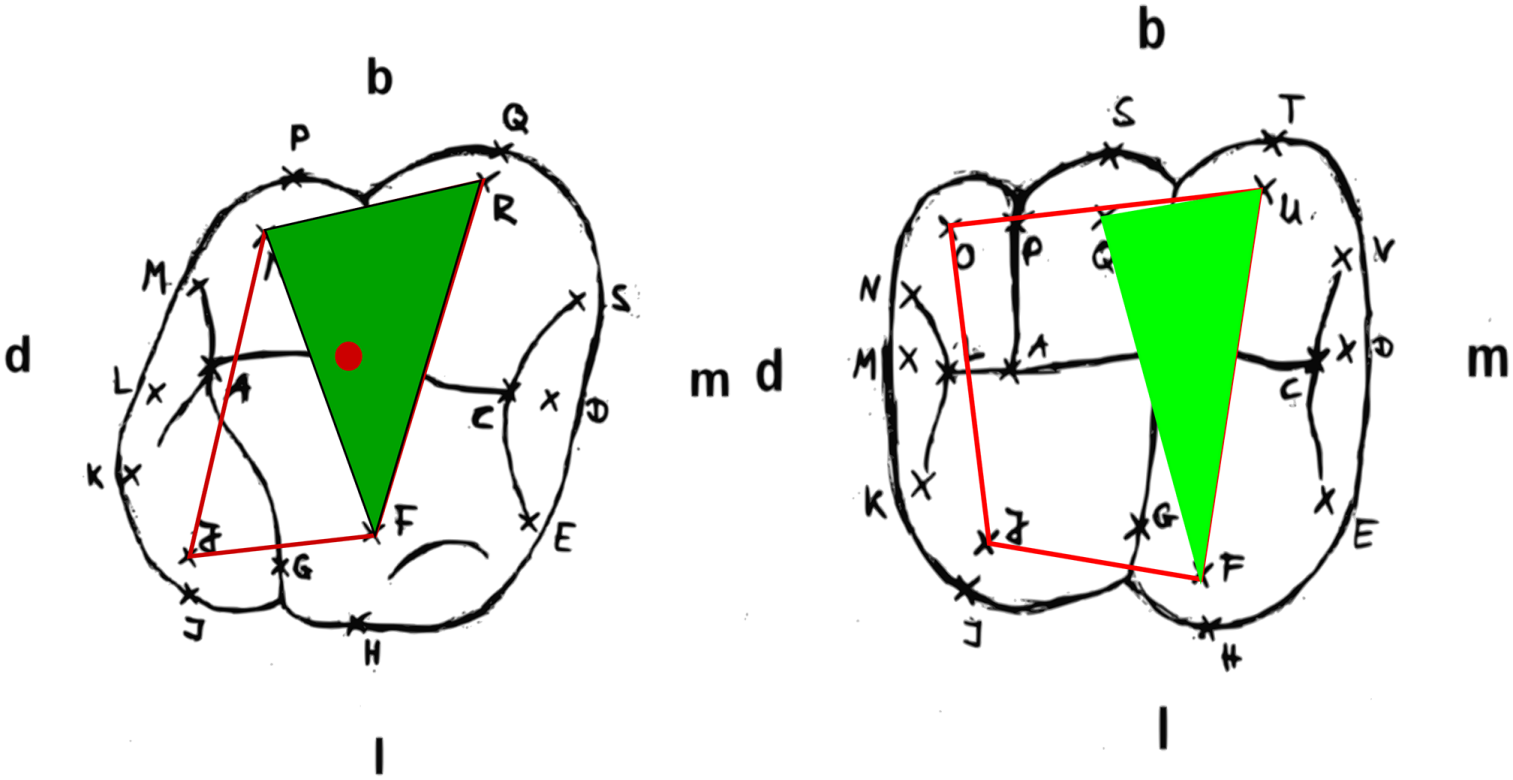
Distances and angles. Measured distances and angles were calculated from 3D coordinates using the software C3D. Orientation: buccal (b), lingual (l), distal (d), mesial (m).

The measured distances and angles were calculated in three dimensions for each tooth. The specific distances and angles are shown in Fig 2 and described in Table 4. The measurement points were selected based on the work of Hartmann [14], Polychronis et al. [17] and Singleton et al. [18]. Distances were defined as connecting lines between cusp tips (occlusal polygon) as suggested by Peretz et al. [19] and Bailey [20].

**Table 4:** Calculated distances and angles.

Maxilla		Mandible	
N_R	Distance from disto-buccal to mesio-buccal cusp	F_J	Distance from mesio-lingual to disto-lingual cusp
R_F	Distance from mesio-buccal to mesio-lingual cusp	U_F	Distance from mesio-buccal to mesio-lingual cusp
F_J	Distance from mesio-lingual to disto-lingual cusp	J_O	Distance from sisto-lingual to disto-buccal cusp
J_N	Distance from disto-lingual to disto-buccal cusp	U_O	Distance from disto-buccal to mesio-buccal cusp
N_R_F [°]	Angle between N_R_F	F_J_O [°]	Angle between F_J_O
B_B1	Distance B perpendicular to area F_R_N	B_B1	Distance B perpendicular to area F_Q_N

The calculated distances represent the mesio-distal and bucco-lingual distance of cusps and their angular relations.

### Geometric morphometry

Geometric morphometry is a collection of methods used to describe and analyze spatial morphological variations in biological structures [21]. Separate individuals and groups can be described and compared based on corresponding measuring points.

First the centroid size was calculated for each tooth with reference to the xyz-coordinates. This is defined as the square root of the sum of the squared distances of the measuring points from the centroid [22]. This enabled the information on size to be preserved. Afterwards, the coordinates were scaled and rotated to a unified coordinate system using Procrustes transformation. The measuring points were then depicted in a scatterplot and compared statistically [23].

Geometric morphometry has already been used in studies within general dentistry and orthodontics. Detailed explanations for this method can be found in ibid [24–26].

To make computation more efficient, a principal component analysis (PCA) was performed. PCA is a procedure for detecting hypothetical variables which explain as much as possible the variance in a multidimensional dataset. The new variables are linear combinations of the original variables.

## Results

### Traditional morphometry—Distances and angles

No differences between the right and left side were found for the measured distances and angles in any of the groups (Table 5). The standard deviations are within a range of 0.2 to 0.8mm and 5° to 9°. Patients with unilateral cleft lip and palate showed no difference between the cleft and non-cleft side (Table 6). For these reasons, the right and left sides were pooled for the subsequent analysis of group differences.

**Table 5:** Right/Left comparison of all groups. T-Test for dependent samples.

		Right		Left		
Group	Distance	Mean [mm]	SD [mm]	Mean [mm]	SD [mm]	p
Maxilla						
European	F_J	4.68010	0.287706	4.60020	0.372217	0.598
	J_N	6.47610	0.702356	6.49740	0.621898	0.944
	N_R	5.30500	0.501562	5.11370	0.653820	0.472
	R_F	6.52110	0.612065	6.42190	0.631132	0.725
	N_R_F [°]	71.42370	4.859986	71.59550	6.788227	0.949
	B_B1	2.45960	0.404881	2.58690	0.615708	0.592
Asian	F_J	4.88033	0.787893	4.86108	0.549092	0.945
	J_N	6.49425	0.664928	6.68617	0.449094	0.416
	N_R	5.42567	0.473951	5.52250	0.627450	0.674
	R_F	7.32525	0.574389	7.16525	0.531215	0.486
	N_R_F [°]	66.71275	6.065928	69.37108	6.962919	0.330
	B_B1	2.39958	0.414803	2.61658	0.524239	0.273
Cleft	F_J	4.53217	0.385757	4.64942	0.273951	0.400
	J_N	6.23875	0.504258	6.27908	0.526210	0.850
	N_R	5.00142	0.553318	5.03350	0.506931	0.884
	R_F	6.42275	0.452926	6.55300	0.447867	0.486
	N_R_F [°]	69.27108	8.981268	69.83525	4.968759	0.851
	B_B1	2.51492	0.316524	2.54875	0.512210	0.847
Mandible						
European	F_J	6.20718	0.615469	6.11200	0.472476	0.688
	U_F	5.42036	0.616578	5.32709	0.400681	0.678
	J_O	4.99020	0.575002	5.14700	0.391181	0.485
	U_O	7.83200	0.537061	7.55950	0.543275	0.274
	F_J_O [°]	73.30918	8.843368	73.93073	6.249277	0.851
	B_B1	2.21027	0.574083	2.28582	0.676576	0.781
Asian	F_J	5.70892	0.508000	5.82400	0.425536	0.554
	U_F	5.91792	0.584342	5.91167	0.561807	0.979
	J_O	5.33967	0.774523	5.46508	0.487950	0.640
	U_O	8.12208	0.620917	7.96733	0.762817	0.591
	F_J_O	70.22233	6.658250	72.89217	5.828841	0.307
	B_B1	2.35950	0.644295	2.41742	0.320292	0.783
Cleft	F_J	5.98279	0.393878	5.80821	0.511353	0.321
	U_F	5.52571	0.443272	5.43750	0.348646	0.563
	J_O	4.98340	0.218020	4.79650	0.404903	0.215
	U_O	7.76140	0.342935	7.51090	0.371617	0.135
	F_J_O	72.76470	6.261608	73.55200	6.675295	0.789
	B_B1	2.37443	0.681399	2.36743	0.583628	0.977

**Table 6:** Comparison of cleft and non-cleft side in the group of cleft patients.

	Non-cleft side		Cleft side		
Distance	Mean [mm]	SD [mm]	Mean [mm]	SD [mm]	p
Maxilla					
F_J	4.60	0.364	4.57	0.313	0.8713
J_N	6.26	0.526	6.25	0.505	0.9632
N_R	5.04	0.557	4.99	0.501	0.8098
R_F	6.46	0.551	6.51	0.331	0.8049
N_R_F [°]	69.95	8.505	69.16	5.732	0.7915
B_B1	2.55	0.337	2.52	0.499	0.8595
Mandible					
F_J	5.93	0.418	5.85	0.504	0.6323
U_F	5.54	0.381	5.42	0.410	0.403
J_O	4.48	0.847	4.51	0.706	0.8256
U_O	7.66	0.625	7.47	0.365	0.3397
F_J_O	73.12	8.357	71.69	6.479	0.622
B_B1	2.38	0.739	2.35	0.506	0.8982

The different groups were compared by performing a t-test for unpaired samples since the measured values presented a normal distribution.

When comparing the study groups (Table 7), no differences in the maxilla were found between the group of Europeans and the group of cleft individuals. However, the mandible exhibited a significantly smaller F_J_O angle in cleft patients.

**Table 7:** Comparison of groups (pooled right and left sides). ANOVA (Mann-Whitney pairwise), asterisks mark statistically significant results.

Distance	Asian [1]		European [2]		Cleft [3]		1–2	1–3	2–3
	Mean	SD	Mean	SD	Mean	SD	p	p	p
Maxilla									
F_J	4.89	0.671	4.64	0.326	4.59	0.332	0.3108	0.0779	0.6041
J_N	6.61	0.557	6.48	0.645	6.25	0.504	0.4943	0.03283 *	0.2924
N_R	5.48	0.556	5.21	0.575	5.01	0.519	0.1284	0.01334 *	0.3896
R_F	7.29	0.513	6.47	0.607	6.48	0.445	0.0003 *	0.00000 *	0.8782
N_R_F	68.04	6.529	71.51	5.746	69.55	7.104	0.0609	0.5028	0.4437
B_B1	2.51	0.475	2.52	0.511	2.53	0.416	0.7954	0.3535	0.629
Mandible									
F_J	5.76	0.462	6.15	0.537	5.89	0.456	0.02562 *	0.08369	0.08369
U_F	5.91	0.560	5.37	0.509	5.48	0.393	0.0020 *	0.01161 *	0.2695
J_O	5.40	0.636	4.92	0.661	4.51	0.706	0.02156 *	0.0000 *	0.06476
U_O	8.04	0.684	7.63	0.553	7.57	0.512	0.07669	0.01927 *	0.7031
F_J_O	71.55	6.269	75.61	4.581	72.41	7.373	0.0490 *	0.9342	0.1885 *
B_B1	2.38	0.498	2.24	0.613	2.37	0.622	0.5165	0.8616	0.4756

Differences in the maxilla of Asians and Europeans was found, with Asians having a significantly increased R_F distance (bucco-lingual width).

### Geometric morphometry

#### Centroid size (CS)

The paired t-test was used to study right/left differences in the centroid size of pooled groups at a given normal distribution (left: p = 0.8538, right: p = 0.1684, Shapiro-Wilk test). No significant differences in the sides were found for the maxilla (CS_left_ = 23.92, CS_right_ = 23.97, p = 0.86726). Likewise, the mandible showed no significant differences between the sides (CS_left_ = 18.9, CS_right_ = 18.96, p = 0.82382).

For patients with cleft lip and palate, no significant difference between the cleft side and non-cleft side was found in the maxilla (CS_cleft_ = 23.2; CS_noncleft_ = 23.4; p = 0.50737) and mandible (CS_cleft_ = 18.4; CS_noncleft_ = 18.6; p = 0.51 529)

**Fig 3:**
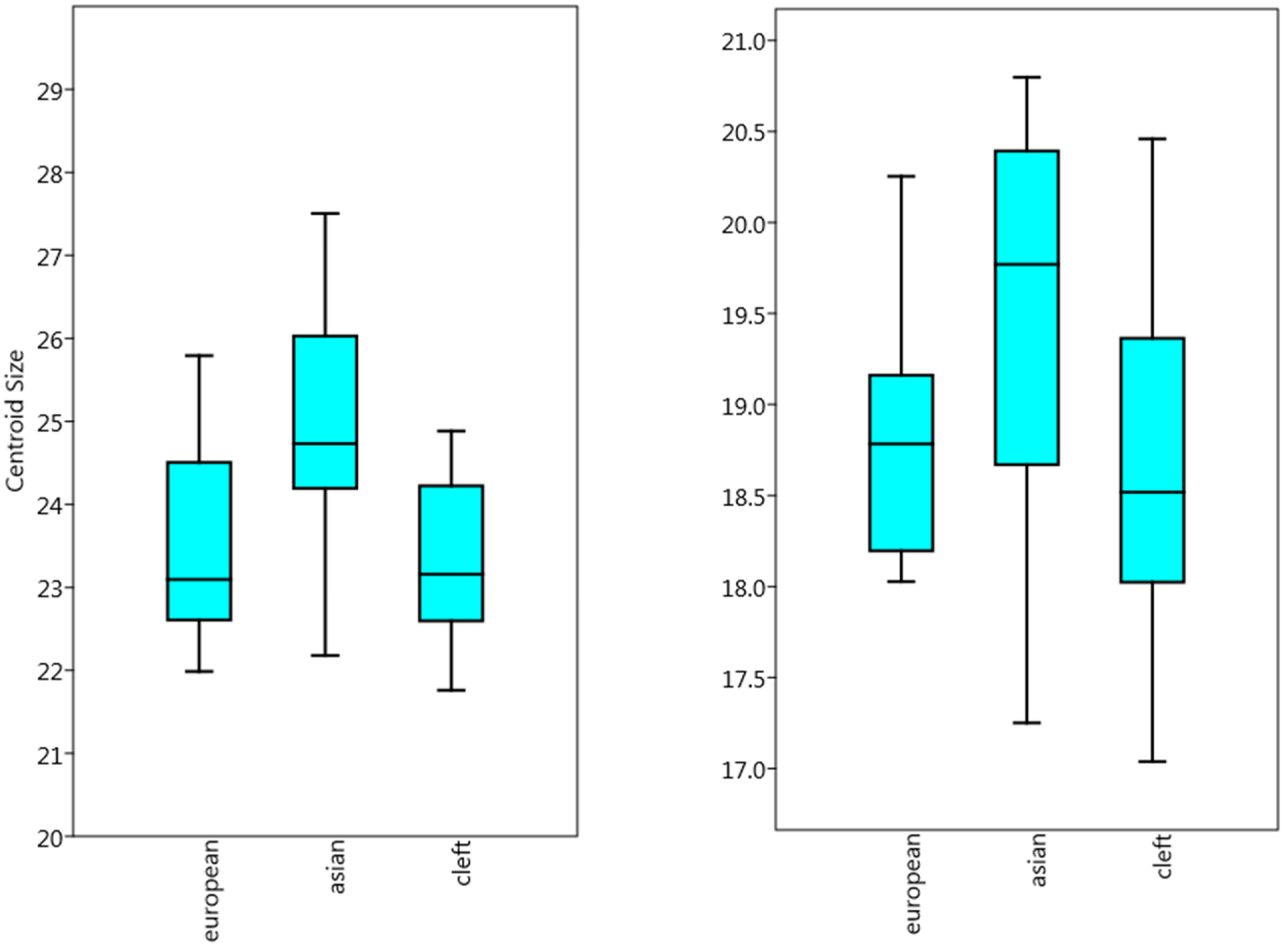
Centroid sizes. Comparison of centroid sizes of maxillary molars (left boxplot) and mandibular molars (right boxplot) in different study groups.

Comparing the three groups (Fig 3), Europeans differed from Asians (p = 0.0022, ANOVA, Mann-Whitney pairwise), and cleft patients also differed from Asians (p = 0.0001). As expected, there was no difference (p = 0.7325) between Europeans and (also European) cleft patients.

The maxillary first molar was significantly larger in the group of Asians. Similarly, there was a significant difference in the mandible between Asians and Europeans (p = 0.03203) and Asians and cleft patients (p = 0.002532). Cleft patients did not differ from non-cleft Europeans (p = 0.3845).

#### Shape

The 3D-coordinates were converted using Procrustes Transformation. Further analysis was performed using PCA. Groups were compared by means of NPMANOVA, a non-parametric test that compares groups on the basis of distances (here: Euclidean distances).

**Fig 4:**
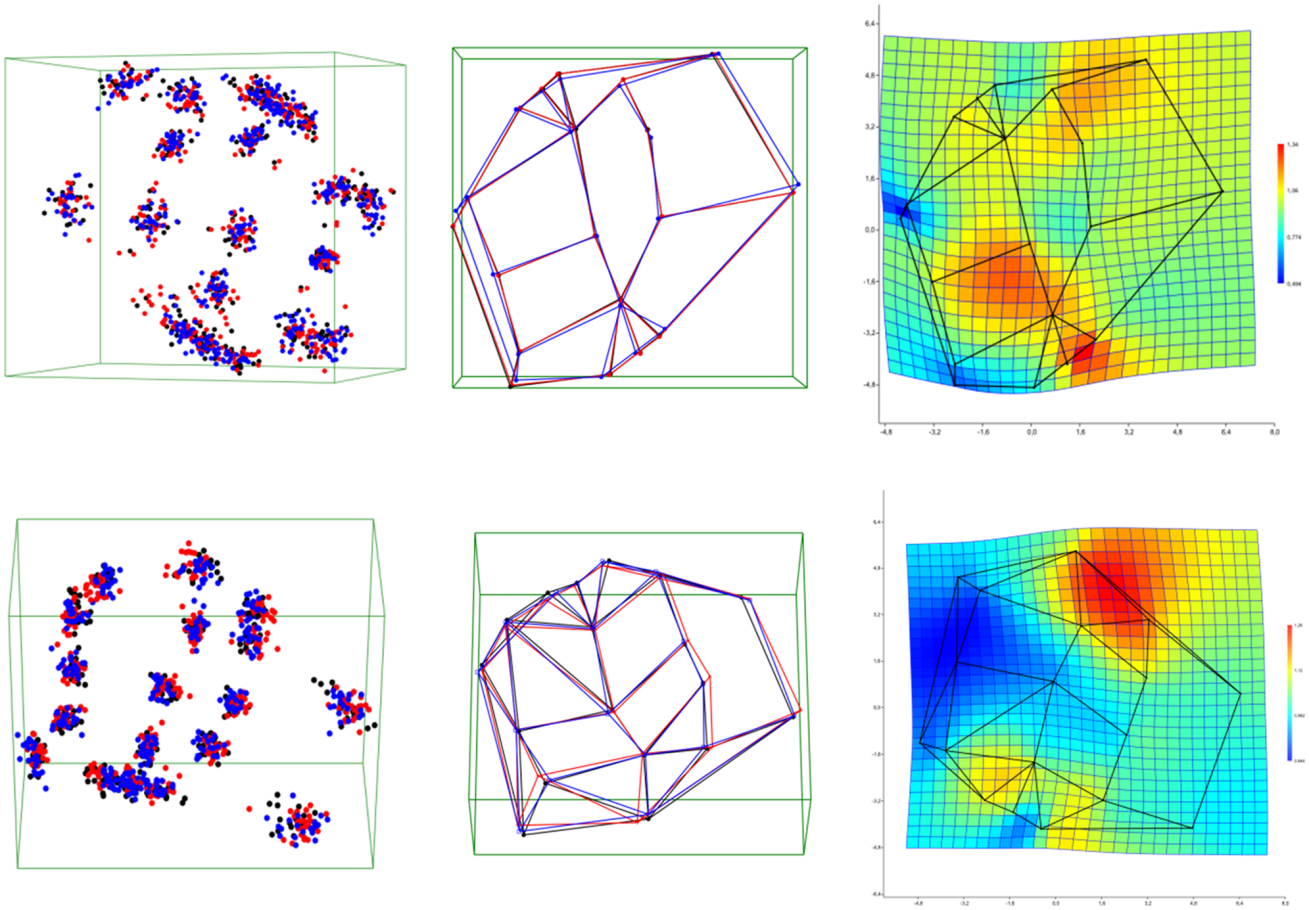
Superimpositions and consensus forms. Left: Superimposition of the transformed coordinates of all maxillary molars (upper row) and mandibular molars (lower row). Color coding: black = Europeans, red = Asians, blue = cleft patients. Middle: Consensus forms of the groups. The comparison illustrates the small differences found. Right: Thin-plate spline deformation in 2D projection (color coding: red = expansion, blue = contraction) compared to the significant differences (maxilla: Asians-cleft, mandible: European-Asian).

The shape of the maxillary molar differs significantly between Asians and cleft patients (p = 0.0011, Bonferroni-corrected, 9999 permutations), but not between Asians and Europeans (p = 0.1169) or Europeans and cleft patients (0.0769). The difference between the group of Asians and cleft patients is mainly due to a mid-distal compression and a slight elevation of the cusp in relation to the gingival portion (Fig 4).

In the mandible, no differences between Europeans with and without cleft formation (p = 0.444) were found; however, the group of Asians differed significantly from both Europeans (p = 0.0009) and Europeans with cleft formation (p = 0.0006). The characteristic morphological difference in Asians is a bucco-lingual extension (Fig 4).

When analyzing the separate groups, no significant right/left differences were found in the maxilla (Europeans p = 0.7105; Asians p = 0.4708; cleft patients p = 0.1688) or in the mandible (Europeans p = 0.162; Asians p = 0.7551; cleft patients p = 0.9842).

Furthermore, the group of cleft patients showed no significant differences in molar shape between the cleft and non-cleft sides (p = 0.2193).

**Fig 5:**
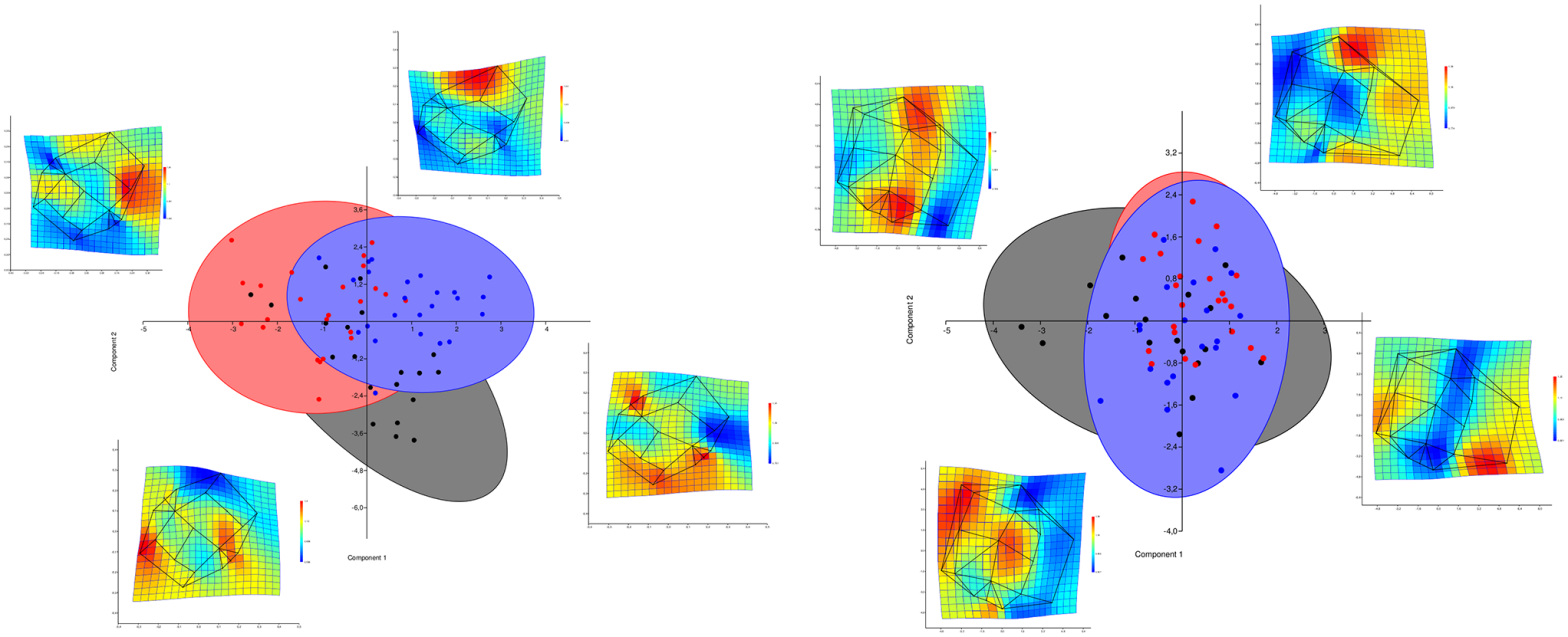
Scatterplot. Scatterplot representing the first two components of the PCA (max = maxilla; mand = mandible; color coding: gray/black = Europeans, red = Asians, blue = cleft patients). However, the first three components only explain 30% of the variance in the maxilla and 34% in the mandible. Additionally, the deforming action of PC1 and PC2 for the maxillary first molar (+1.5 to -1.5) and mandibular first molar (+2 to -2) is shown in the 2D projection as a thin-plate spline (color coding: red = expansion, blue = contraction).

In the principal component analysis (PCA), the first six components are considered relevant (broken stick in scree plot at 6, Fig 5). The variance in shape in relation to the axis of the main components (principal component, PC) is shown in Table 8.

**Table 8:** Shape variances corresponding to each PC axis for maxillary and mandibular first molars.

Principal Component	Maxillary molar		Mandibular molar	
	Variance (%)	Cumulative Variance (%)	Variance (%)	Cumulative Variance (%)
1	18.8	18.8	15.5	15.5
2	11.2	30	10.9	26.4
3	10.6	40.6	9.0	35.4
4	8.8	49.4	7.7	43.1
5	6.5	55.9	7.5	50.6
6	5.8	61.7	4.6	55.2

## Discussion

### Critique of methodology

The low number of models in this study can be attributed to the high occurrence of fissure sealants and occlusal fillings, especially in the group of cleft patients. Moreover, cleft patients exhibit a high prevalence of caries and insufficient vertical development with abrasions of M1 [27]. Therefore, owing to the strict inclusion criteria, only 16 models were selected from a sample of 450 cleft patient models.

The prevalence of tooth wear is also high in children and adolescents: 30% of 14-year-old school children show abrasions with exposure of dentin [28].

The gender distribution in each study group is unbalanced, but consistent between the groups. Nevertheless, any identified gender differences should be interpreted with caution. Statements on sexual dimorphism of teeth are inconsistent. The teeth of male individuals are about 2–6% larger on average [29]. However, according to previous studies, only the canines consistently show features of sexual dimorphism [12]. No differences in molar shape were found between genders [30]. Evaluating female and male teeth as one group rather than separately based on gender can consequently be justified.

### Characterization of groups based on orthodontic metrics

The measurement values for the group of Europeans were consistent with the expected average values of a population sample. Overbite and overjet were slightly increased, as was the prevalence of Angle Class II relationships, since some models were taken from patients prior to orthodontic treatment [31]. An enlarged middle transverse width was striking in the group of Asians. In contrast, the group of cleft patients showed significant sagittal (overjet) and transverse deficiencies. The high standard deviations in the group of cleft patients, particularly in the maxillary anterior dental arch width, are attributable to the highly individual characteristics of cleft formation [32]. It should be noted that orthodontic pre-treatment in the group of cleft patients had been done with removable appliances only.

### Traditional morphometry

Reflex microscopy is a contactless, three-dimensional coordinate measurement that uses a projected laser spot. It has a measurement error of less than 0.15 mm [33]. Previous studies have proven the suitability of using reflex microscopy on models with cleft lip and palate [34–36] and for measuring tooth morphology [37].

The measurement of distances and angles showed no significant left-right differences. In cleft patients, the asymmetry of the anterior tooth widths was associated with the general instability of development [38]. A differentiation of the cleft/non-cleft sides (Table 6) showed no abnormalities. When comparing the ethnic groups in this study, significant differences were found, particularly in the mandible. Asians were found to have larger teeth in all dimensions, especially in the mandible. Our results therefore suggest that ethnicity may affect tooth width. Its influence on the dental arch form and size has already been reported in the literature [39,40]. As a result, analyses that are based on the comparison of different tooth widths are not transferable to other populations [41]. This corresponds to the results of Hasegawa et al. [42].

### Geometric morphometry—Centroid size

The group of Asians presented a significantly greater centroid size in both the maxilla and the mandible. This could be due to an actual enlargement (which also corresponds with clinical experience) or it could be influenced by the unequal gender distribution. The identified differences support the studies of Endo et al. [41] and Hasegawa et al. [42].

### Geometric morphometry—Shape

Methods of geometrical morphometry provide considerably more information than linear measurements [43]. In this study, the entire morphology of the crown was mapped by measuring points. This method, based on molar morphology, offers significant advantages for detecting similarities and differences between population groups.

No signs of directional asymmetry of the maxillary or mandibular molars were found in this study. This corresponds to the findings of Noss et al. [44]. Furthermore, no differences in molars were detected with regard to the cleft side. Thus, nonlocal mechanisms seem to be responsible for dental anomalies in cleft patients [45].

### Cleft lip and palate and first molars

Kraus et al. [46] described numerous anomalies, including shape abnormalities, in maxillary and even mandibular teeth associated with cleft formation. Animal experiments also confirm an association between MSX1 mutation, orofacial clefts and aplasia of teeth [45]. Despite these references to additional complex effects, the present study suggests that the process of cleft formation (7^th^ - 11^th^ week) has no local or temporal effect on the development of first molar crowns which occurs later (initial calcification of M1: 28^th^ - 32^nd^ week). Size reduction and asymmetry, as described by Sofaer [38] and Werner & Harris [47], could not be confirmed. However, these studies were only based on the mesiodistal crown diameter and the sample sizes were larger.

### Questions & answers

1. Are there any characteristic differences in the size and shape of first molars in the maxilla and mandible between Europeans, Asians and Europeans with unilateral cleft lip and palate? *Differences were found involving larger crown dimensions in Asians and altered sagittal-transverse relationships*.
2. Are there differences between the right and the left side of the dental arch?
3. *There was no evidence of side-to-side differences based on the available data*.
4. Does unilateral clefting influence the morphology of molars?

A unilateral cleft does not seem to affect the morphology of molars.

## Conclusion

Unilateral clefting did not affect the size and shape of molars. By contrast, ethnic differences in the size and shape of teeth were confirmed.

The results are relevant for orthodontic treatment that uses preadjusted appliances and prosthetic CAD/CAM restorations.

Most modern orthodontic multibracket-appliances are preadjusted to a set “average” tooth morphology. In patients with a tooth morphology that does not correspond exactly to the one the appliance was programmed for, individual adjustments are imperative in order to align the teeth in an ideal position. From this perspective, the results support the use of fully customized CAD/CAM manufactured appliances in orthodontics. In terms of the automatic generation of restorations, ethnic differences should be considered and the morphologies proposed by algorithms that are based on a particular ethnic group should not be applied uncritically to another ethnic group.

However, the lack of evidence of side-to-side differences does allow the possibility of mirroring crowns or brackets/attachments in a CAD/CAM workflow.

## Supporting information

S1 Table Patient characterization based on orthodontic metrics. (CSV)

S2 Table Traditional morphometry maxilla. (CSV)

S3 Table Traditional morphometry mandible. (CSV)

S4 Table Centroid size maxilla. (CSV)

S5 Table Centroid size mandible. (CSV)

S6 Table Coordinates PAST maxilla. (CSV)

S7 Table Coordinates PAST mandible. (CSV)
